# Modulation of Microglial Function by ATP-Gated P2X7 Receptors: Studies in Rat, Mice and Human

**DOI:** 10.3390/cells13020161

**Published:** 2024-01-16

**Authors:** Manju Tewari, Stephanie Michalski, Terrance M. Egan

**Affiliations:** Department of Pharmacology and Physiology, and The Henry and Amelia Nasrallah Institute for Translational Neuroscience, Saint Louis University School of Medicine, St. Louis, MO 63104, USA; stephanie.michalski@health.slu.edu (S.M.); terrance.egan@health.slu.edu (T.M.E.)

**Keywords:** ATP, microglia, purinergic receptors, P2X7, innate immunity, tumor microenvironment, membrane permeabilization, CNS diseases

## Abstract

P2X receptors are a family of seven ATP-gated ion channels that trigger physiological and pathophysiological responses in a variety of cells. Five of the family members are sensitive to low concentrations of extracellular ATP, while the P2X6 receptor has an unknown affinity. The last subtype, the P2X7 receptor, is unique in requiring millimolar concentrations to fully activate in humans. This low sensitivity imparts the agonist with the ability to act as a damage-associated molecular pattern that triggers the innate immune response in response to the elevated levels of extracellular ATP that accompany inflammation and tissue damage. In this review, we focus on microglia because they are the primary immune cells of the central nervous system, and they activate in response to ATP or its synthetic analog, BzATP. We start by introducing purinergic receptors and then briefly consider the roles that microglia play in neurodevelopment and disease by referencing both original works and relevant reviews. Next, we move to the role of extracellular ATP and P2X receptors in initiating and/or modulating innate immunity in the central nervous system. While most of the data that we review involve work on mice and rats, we highlight human studies of P2X7R whenever possible.

## 1. Introduction

Nucleotide triphosphates play many roles in the everyday physiology of all cells. They are vital components of the genetic code, essential cofactors for countless enzymatic reactions, and fundamental phosphate donors in biosynthesis [1]. Adenosine triphosphate (ATP) is particularly important because it functions both inside and outside the cell. Intracellular ATP provides the power needed to drive a vast array of energetically unfavorable chemical reactions within cells, whereas extracellular ATP (eATP) and its metabolites activate plasmalemmal purinergic receptors that initiate and modulate cellular function [2,3]. The purinergic receptor superfamily contains three branches that differ in structure, function, and pharmacology [4,5]. The first class, called P1 receptors (more commonly called “A receptors”), are metabotropic receptors activated by nanomolar to low micromolar concentrations of extracellular adenosine, a common by-product of dephosphorylation of eATP. Four subtypes (A1, A2A, A2B, and A3 receptors) are coupled to G-proteins, and microglia express all four [6,7,8,9,10,11,12]. The second class of purinergic receptors, called P2Y receptors (P2YRs), resemble adenosine receptors in signaling through the activation of G-protein coupled receptors. This subfamily is composed of eight mammalian subtypes (P2Y1R, P2Y2R, P2Y4R, P2Y6R, P2Y11R, P2Y12R, P2Y13R, and P2Y14R), many of which are expressed in microglia [13,14,15]. Individual family members have a preferred native agonist of ADP, ATP, UDP, or UTP [16]. P2YRs play essential roles in microglial chemotaxis, phagocytosis, and the release of pro-and anti-inflammatory cytokines [17,18,19,20].

### P2X Receptors

P2X receptors (P2XRs) form the third class of purinergic receptors. They are a family of seven ATP-gated ion channels, designated P2X1R–P2X7R, that depolarize cells by increasing membrane permeability to Na^+^, K^+^, and Ca^2+^ [21]. In humans, five of the seven subtypes form homotrimeric complexes capable of stimulating transmembrane current in response to eATP. Human P2X5R and P2X6R are the exceptions. In the case of human P2X5R, a single-nucleotide polymorphism in p2rx5 prevents transcription of the exon 10 resulting in an incomplete protein that lacks a part of the pore-lining second transmembrane domain (TM2); the resulting product properly traffics to the surface membrane but fails to respond to eATP [22]. When engineered to contain all 13 exons, the transfection of cDNA encoding the full-length human P2X5R results in the expression of functional eATP-gated receptor which, unlike other family members, displays significant Cl^−^ permeability (i.e., relative Cl^−^ to Na^+^ permeability equals 0.5 for human P2X5R as compared to <0.1 for other P2XR subtypes) [23]. While the expression of the functional, full-length P2X5R does not normally occur in humans, a single G > T polymorphism would make it possible, an outcome worth considering when studying the effects of eATP on human immune cells that show abundant levels of P2X5R mRNA [22].

Like the case of human P2X5Rs, the expression of the gene encoding the human P2X6R also does not result in a functional receptor. The lack of ATP-gated current reflects the fact that monomeric P2X6 subunits are retained in the endoplasmic reticulum and require a partner (either P2X1, P2X2, or P2X5) to shuttle it to the plasma membrane as a heteromeric receptor [24,25]. Indeed, the ability to form heteromeric receptors is a common feature of most P2XRs [26], including those found in native tissues [27]. The P2X7 receptor is the only receptor for which this is not true. It is incapable of pairing with other P2XR subtypes and always expresses as homotrimeric P2X7R [26,28]. There are 11 P2X7R rodent splice variants (P2X7aR–P2X7kR) and eight human variants (P2X7AR–P2X7HR), of which only P2X7AR and P2X7BR form functional complexes gated by eATP in humans [29]. P2X7BR differs from P2X7AR in four ways: it shows higher mRNA transcript numbers; it has a truncated C-terminal cytoplasmic domain; it does not induce membrane permeabilization (see below); and, it is less sensitive to eATP [30]. Co-expression of P2X7AR and P2X7BR produces a leftward shift in the eATP concentration–membrane current response curve and increased uptake of fluorescent dyes, demonstrating that the co-assembly of the two splice variants into a single complex results in a heteromeric P2X7A/P2X7B receptor with enhanced function [31].

P2X7ARs are unique in other ways too. Chief amongst these differences is a low sensitivity to eATP by comparison to other family members, absence of desensitization, and persistent current facilitation in response to sustained agonist ligation [32]. Like all functional P2XRs, the P2X7aR is composed of three subunits, with each subunit spanning the membrane twice. The N- and C-termini are intracellular, and the orthosteric eATP-binding site is formed at the extracellular interface of adjoining subunits [33,34]. The C-terminus is ~200 amino acids longer than those of P2X1R–P2X6R and has sites that bind accessory proteins, modulate receptor function via post-translational modification, regulate desensitization, and help target the receptor to the surface membrane [30,35,36,37]. Recent structural data obtained from single-particle cryo-electron microscopy were the first to describe the complete structure of a full-length wild-type P2X7R with intact cytoplasmic domains, and this landmark paper from the Mansoor laboratory provides plausible molecular explanations for two of the unique properties mentioned above [34]. First, unlike other P2XRs, the long C-terminus of the P2X7aR contains a unique 18-amino acid cysteine-rich domain that the authors call the “C-cys anchor”, which acts as a molecular hinge that prevents desensitization by using palmitoyl groups to anchor the pore-lining TM2 and the cytoplasmic domains to the inner leaflet of the lipid membrane. This lack of desensitization imparts P2X7aRs with the profile of sustained application that is a hallmark of P2X7aR’s pharmacological fingerprint. Second, P2X7aRs show an unusually low affinity for eATP, which traditionally has been explained by assuming that fully ionized ATP (i.e., ATP^4−^), a form of the nucleotide that is in low abundance in the presence of the millimolar concentrations of divalent cations (predominately Ca^2+^ and Mg^2+^) normally present in interstitial fluids, is the natural agonist [34,38]. Now, the solved structure offers another possibility: the entrance to the extracellular ligand binding pocket of the P2X7 receptor is narrow in comparison to other subtypes, suggesting that the low affinity of eATP for P2X7aRs might result, in part, from limited access to its binding site [34]. This hypothesis could be directly tested by comparing the atomic scale structures of the P2X7aR to the “k” splice variant of murine P2X7Rs (i.e., murine P2X7kR) that show a remarkable eight-fold difference in agonist sensitivity [39].

Human tissues do not express the more sensitive “K” splice variant, and therefore, relatively high concentrations of eATP are needed to trigger a P2X7AR-mediated response in humans. The low sensitivity of the P2X7AR (the main focus of this review and henceforth simply referred to as “P2X7R”) is physiologically significant because it provides a means by which eATP can act as a danger-associated molecular pattern (DAMP) that signals cell damage. The extracellular concentration of eATP is low (~10 nM) in healthy tissue because of limited release and rapid hydrolysis by ectonucleotidases [40,41,42]. However, it rises when cytoplasmic ATP (~1–10 mM) is released through canonical (exocytosis) and non-canonical (cell lysis and passive transport through ion channels) pathways [43,44]. Any condition that permeabilizes membranes results in the outward leak of intracellular ATP [42,45]. The result is an increase in eATP at sites of inflammation and tissue damage [46] that, for example, can reach concentrations as high as hundreds of micromolar in the microenvironment of tumors [47,48,49]. In the CNS, multiple pathologies, including ischemia, trauma, oxidative stress, hypoxia, neurodegenerative insult, cellular stress, and epilepsy, significantly elevate concentrations of eATP and its metabolites in the interstitial fluid [50]. Low concentrations of eATP (tens of micromolar) act as short-range “find me” signals directing motile phagocytes to damaged tissue by activating metabotropic P2YRs [51,52,53,54,55]. Higher concentrations (hundreds of micromolar) activate P2X7Rs, which in some cases result in the translocation of phosphatidylserine, bestowing an “eat me” signal in the outer leaflet of the plasma membrane that targets cells for engulfment and cell death [56,57,58]. Interestingly, the activation of P2X7Rs does not trigger phosphatidylserine translocation or phagocytosis in cultured human microglia. Instead, eATP decreases the uptake of fluorescently labeled *E. coli* bioparticles [59], which supports the hypothesis that the closed/inactive conformation of the P2X7R acts as a scavenger receptor for innate phagocytosis in the absence of eATP [60].

One final feature sets P2X7Rs apart from other members of the P2XR family. Some cells, particularly those associated with the immune system, express a P2X7R that does not initiate a measurable response to the application of eATP [61,62]. nfP2X7R (“nf” for non-functional) is normally retained intracellularly and released to the membrane upon stimulation with concentrations of eATP > 0.5 mM. It is expressed in a wide range of cancers where it promotes tumor cell survival [62,63]. Relatively little is known about how this unique receptor works, so further work is warranted. To date, there are no reports of expression of nfP2X7R in microglia of any species.

## 2. Microglia

The human brain contains ~160 billion cells. Roughly half of these are neurons, with the remainder comprised of endothelium (~20 billion) and neuroglia [64]. Originally, neuroglia were thought to simply provide physical and nutritional support to neurons [65]. However, in light of an expanding body of evidence, it is now abundantly clear that these cells play substantive roles in neural development, plasticity, homeostasis, and disease [20,66,67].

Approximately 5–20% of the ~60 billion glial cells in the human brain are microglia [68]. Microglia begin life as erythromyeloid progenitor-derived yolk sac macrophages that populate the nervous system during early embryogenesis [67,69]; the idea that fetal-liver-derived monocytes also contribute is postulated but controversial [70]. In humans, migration begins at ~4.5 gestational weeks, with a second wave occurring ~8 weeks later [71,72]. The precise route by which they enter the CNS is unknown but may involve the meninges, ventricles, and vasculature [67,73]. Microglia colonize the CNS in a grid-like fashion and quickly develop phenotypes that vary as a function of the soluble components of CNS microenvironments [74,75]. These environmental signals are sensed by a family of microglial proteins collectively known as the “sensome”, which includes receptors for pattern recognition signals, danger-associated molecular patterns (DAMPs), Fc fragments, chemokines, cytokines, and extracellular matrix proteins [76]. Purinergic receptors, including the P2X7R, form a significant portion (~8%) of the sensome, highlighting the importance of eATP and its metabolites in directing microglial function. Regional differences in postnatal microglial are reported in rodents [77,78,79,80,81] and humans [82,83,84] but remain controversial [67,75]. For example, the ability of eATP stimulation of microglia to kill co-cultured neurons depends in part on the section of the brain from which the microglia were harvested [77]. In addition, microglia show developmental differences in regional densities, transcriptomes, activation states, and functional properties that vary between males and females [85,86,87,88], including humans [82,89].

In postnatal infants and healthy adult animals, an intact blood–brain barrier prevents further invasion by components of the peripheral immune system, and microglia depend on proliferation and apoptosis to sustain a relatively stable population of mature cells [90,91,92]. Absolute rates of proliferation are controversial [93] but appear to vary by brain region [94]. The average lifespan is thought to be ~4.2 years in humans, with about 2% of the total microglial population proliferating at any one time [91,95]. It is worth noting that many diseases compromise the blood–brain barrier; glioblastoma is an example. In these cases, peripheral blood monocytes and non-parenchymal macrophages migrate into the CNS, where they differentiate into cells that can be hard to distinguish from resident microglia [74,75,96,97,98]. Further, serum permeates the compromised blood–brain barrier, exposing the CNS parenchyma to regulatory signals that it normally would not encounter [99]. Because it promotes cell division [100], the leak of serum into the CNS environment might explain the increased microglial proliferation and phagocytosis observed following stroke and trauma [100].

Microglia express a common pool of developmental and homeostatic genes in a wide range of species, including humans. However, unlike other animals, human microglia show significant heterogeneity with clearly delineated subsets that are unrelated to sex [101]. Further, humans express a greater number of genes conferring susceptibility to Alzheimer’s and Parkinson’s diseases than mice and rats (see below). Thus, although procurement of tissue samples of the human brain is problematic and the range of possible in situ experimental manipulations is severely limited by comparison to experiments on rodents, more data from work on human tissue is needed to better understand how microglia contribute to human CNS disease [102].

### 2.1. Microglia Express Multiple Subtypes of Purinergic Receptors

Microglia express key elements of the “purinome” [103] and are capable of releasing, metabolizing, and sensing ATP [15,104]. They express a number of ectonucleotidases, including CD39, which metabolizes ATP and ADP to AMP; NPP1, which metabolizes ATP to AMP; and CD73, which metabolizes AMP to adenosine [105,106]. Microglia also express relatively high mRNA levels for metabotropic (adora2A, adora3, p2ry2, p2ry6, p2ry12, and p2ry13) and ionotropic (p2rx4 and p2rx7) purinergic receptors [15,107,108]. The additional receptors identified by functional studies include A1, A2A, P2X1, P2Y1 and P2Y14 receptors [109,110,111,112]. The relative abundance of each receptor subtype depends in part on the sex of the animal [86] and the activation state of the microglia [110]. For example, seizures in a rodent model of epilepsy cause the upregulation of mRNA expression for P2Y6Rs, P2Y12Rs, P2Y13Rs, P2X4Rs, and P2X7Rs in hippocampal microglia [113].

It is difficult to assign distinct physiological responses to specific purinergic receptors. However, some generalizations hold true. Adenosinergic A3Rs regulate microglial process extension and migration of microglia [11,114]. Metabotropic P2Y6Rs underlie phagocytosis [115], and P2Y12Rs mediate cell migration [15,52]. Ionotropic P2X4Rs [116,117] and P2X7Rs [118,119,120] are linked to neuropathic pain. Additionally, P2X7Rs mediate the release of proinflammatory cytokines in response to tissue damage and inflammation [108,121,122].

### 2.2. Healthy Prenatal CNS

Microglia, under the direction of the chemotactic receptor CX3CR1 [69,123,124], infiltrate the CNS as neuronal circuits begin to assemble [71,125] and play essential roles in the development of the nervous system [126,127,128]. In rats and monkeys, they selectively colonize proliferative zones during the late stages of cortical neurogenesis and limit the number of neurons by phagocytosing precursor cells [129]. Further, they influence the differentiation of both astrocytes and oligodendrocytes in mice [72,130] and humans [72] and may be angiogenic [131,132]. Colonization is dimorphic, with sex-dependent differences in regional densities that vary with time [133,134]. For example, male and female rodents have equivalent densities of activated microglia in the amygdala, hippocampus, and parietal cortex at birth. However, density is higher in males in comparison to females at postnatal day 4, and higher in females than males in mature adults [85]. The chemotactic signals that attract microglia to the brain are incompletely characterized but certainly include CX3CL1 (also known as fractalkine). Genetically modified mice lacking the fractalkine/CX3CR1 receptor show a significant reduction in the number of microglia in the brain and a concurrent increase in the density of dendritic spines, suggesting that CX3CR1 guides the migration of the microglia responsible for pruning synapses in early development [123,124,135]. Nucleotides also play a role, as the high concentration of extracellular nucleotides that accompanies developmental neuronal apoptosis is suggested to act as a chemotactic signal for recruitment of microglial precursors into zebrafish brain [136]. The identity of the responsible nucleotide is unknown but probably derived from ATP, which acts through metabotropic P2Y12Rs to direct microglia to sites of CNS injury in adult murine brains [52,137].

### 2.3. Healthy Postnatal CNS

Immature microglia invade the CNS as irregular-shaped cells with blunt processes, a morphological profile that resembles activated microglia in adults [89,138,139,140]. They disburse in a uniform fashion, with typical cell-to-cell distances of about 50–60 µm in adult mice [141] and remain in place in the absence of a migratory signal or pathophysiological insult [142]. Their amoeboid shape changes by postnatal day 10 to a ramified morphology of radial extensions from a small rod-shaped cell soma, with a full extension completed by postnatal day 28 [73]. The first-order branches, which can reach a length of 50 µm, are highly motile, extending and retracting at a rate of 1.5 µm/min and capable of surveying the entire brain environment once every few hours [141,143]. Normal process extension and baseline surveillance depends on tonic activation of the two-pore K^+^ channel, THIK-1, which is responsible for setting the resting membrane potential in mouse microglia [19]. Thinner filopodia spread from the tips of the larger branches and extend and retract in a manner that is independent of THIK-1 but modulated by cAMP, enabling precise surveillance of the immediate environment under spatiotemporal control of intracellular cAMP microdomains [144]. Purinergic receptors also come into play in human brain slices, as low doses of ADP trigger process extension through the activation of P2Y12Rs and higher doses trigger process retraction through the activation of P2Y1Rs and P2Y13Rs [112]. Adenosine, a by-product of ATP metabolism, also triggers process retraction by activating Gs-protein-coupled A2A receptors [8].

Although microglia contact a variety of cell types, they preferentially target active neurons [145,146,147]. Approximately 90% of neocortical neurons in mice and humans are in contact with a microglial process at any one time, with typical lifespans of 7.5 and 25 min for dendritic and somatic connections, respectively [148]. Interestingly, somatic connections display a unique molecular architecture of apposing clusters of neuronal Kv2.1 K^+^ channels and microglial P2Y12 receptors, a pattern not seen in dendritic contacts. Neuronal mitochondria, vesicular nucleotide transporter (vNUT), and an ectonucleotidase (NTPDase1) are positioned close to the Kv2.1 clusters, suggesting that neurons signal increases in activity by triggering the vesicular release of mitochondrial-derived ATP, which is rapidly converted to ADP, the natural agonist for the P2Y12R [148].

As the primary immune cell of the CNS, microglia attack infections of the brain and spinal cord (see below). However, in the absence of infection, microglia remain active and play critical roles in synapse maturation and remodeling [149]. Microglia express and release neuroprotective chemicals that promote the development of glial precursor cells, neurons, and oligodendrocytes [138]. At the same time, neurons upregulate the expression of fractalkine at peak times of synapse maturation (P15 in mice), providing a strong signal for the migration of microglia to developing synapses [150]. Once in place, microglia engulf and phagocytose dendritic spines to reduce weaker afferent input and fine-tune synaptic connectivity [135]. In mice, immature synapses formed of inactive afferent fibers are opsonized by complement proteins C1q and C3 [151,152], selectively targeting them for engulfment by resident microglia [146,149,153,154]. Phagocytosis of synapses continues into adulthood, as healthy adults show selective elimination of newly formed synapses during REM sleep [155,156,157], which might be explained by the suppressive effects of the circadian release of noradrenaline and glucocorticoids on microglial surveillance during waking hours [158]. Further, recent work suggests that complement-dependent synapse elimination by microglia leads to the forgetting of previously learned contextual fear memory in adult mice [159]. At the same time, microglia strengthen active synapses by secreting neurotrophic factors such as insulin-like growth factor, nerve growth factor and brain-derived neurotrophic factor [142,160,161], promote the synchronized discharge of neighboring neurons [162], and play a critical role in learning and memory [163,164].

### 2.4. Infection and Disease

Microglia are the first line of defense against bacterial, fungal, and viral infections of the CNS [165]. They respond to the detection of highly conserved microbial motifs, known as pathogen-associated molecular patterns (PAMPs), with a change in morphology and up-regulation of defensive signaling pathways. Common PAMPs include components of bacterial cell walls such as lipopolysaccharides (LPS) from Gram-negative bacteria, lipoteichoic acid from Gram-positive bacteria, and double-stranded RNA from viruses [166]. PAMPs are recognized by Toll-like receptors (TLRs), a family of pattern recognition receptors (PRRs) found in the cytosol and membranes of immune cells [167], including the human microglia [168,169]. Specific PAMPs are recognized by specific PRRs, with bacterial lipoteichoic acid and fungal zymosan recognized by TLR2, viral dsRNA by TLR3, bacterial LPS by TLR4, and bacterial flagellin by TLR5 [170,171]. The activation of TLRs promotes NF-κB-dependent signaling cascades, resulting in the production of chemokines (MIP-2, MCP-1), proinflammatory cytokines (TNF-α, IL-1β), and reactive oxygen/nitrogen species [170]. In addition, infection begets inflammation, triggering the release of DAMPs such as eATP from aggravated tissues, which promote the migration of phagocytes to the site of injury. The final outcome is the elimination of the insult through programmed cell death and phagocytosis [70,167,172]. Interestingly, new evidence from engraphment studies suggests that reactive microglia retain the ability to revert to the homeostatic ramified state when placed in a permissive environment lacking activation signals, suggesting that microglia are capable of surviving infection and reestablishing active surveillance upon the removal of the offending PAMP/DAMP [74].

Microglia express genes tied to neurodegeneration [173], and microglial expansion and activation are common features of neurodegenerative diseases [174,175,176,177]. Amongst these cells is a subset originally identified in rodent models of Alzheimer’s disease, amyotrophic lateral sclerosis, multiple sclerosis, and aging [178] but present in humans too [179,180]. Called disease-associated microglia (DAMs), they show downregulation of homeostatic signature genes such as cx3cr1, purinergic p2ry12, and tmem119, and upregulation of genes associated with lysosomal, phagocytic, interferon response, and lipid metabolism pathways [70]. The conversion of resting ramified microglia to activated amoeboid DAMs involves the recognition of neurodegeneration-associated molecular patterns (NAMPs) present on damaged and dying neurons, extracellular protein aggregates, and products of lipid degradation [178]. The signaling pathways responsible for the change in phenotype are under investigation, with good evidence supporting a role for the Triggering Receptor Expressed on Myeloid Cells 2 (TREM2) in mediating increases in proliferation and phagocytosis that define reactive microgliosis [181]. For example, genetically modified rats lacking TREM2 fail to show the DAM phenotype [182]. Further, TREM2 binds oligomers of β amyloid, leading to the colocalization of DAMs and plaques in a rodent model of Alzheimer’s disease [183,184,185,186]. Colocalization provides a protective shield that prevents neurons from exposure and positions microglia close to fibrillar plaques to facilitate phagocytosis [175]. Lastly, microglia express and release Apolipoprotein E (ApoE), a putative TREM2 agonist, and polymorphisms in the Apoe gene are a major risk determinate of Alzheimer’s disease [187,188]. Apoe is upregulated in DAMs, suggesting that the agonist, ApoE, and its receptor, TREM2, form a positive feedback loop that facilitates transition to the reactive DAM phenotype [175,189].

## 3. P2X7R and Microglia

Microglia show constitutive expression of p2rx4 and p2rx7 in both rodents and humans [20,108]. Microglial P2X4Rs play critical roles in rodent models of neuropathic pain [117,190], experimental allergic encephalomyelitis [191,192], and chronic migraine [193]. In keeping with these studies, relatively low concentrations of eATP (≤100 µM) evoke desensitizing non-selective inward currents and sustained outward K^+^ currents in murine microglia that most likely result from activation of P2X4Rs and P2Y12Rs, respectively [18,19,194,195,196,197]. Surprisingly, these same concentrations of eATP fail to trigger inward membrane current or Ca^2+^ influx in cultured human microglia, suggesting that either the p2rx4 is downregulated in culture, the mRNA is not translated, or the properly translated protein is not trafficked to the cell surface membrane [59,198].

**Figure 1:**
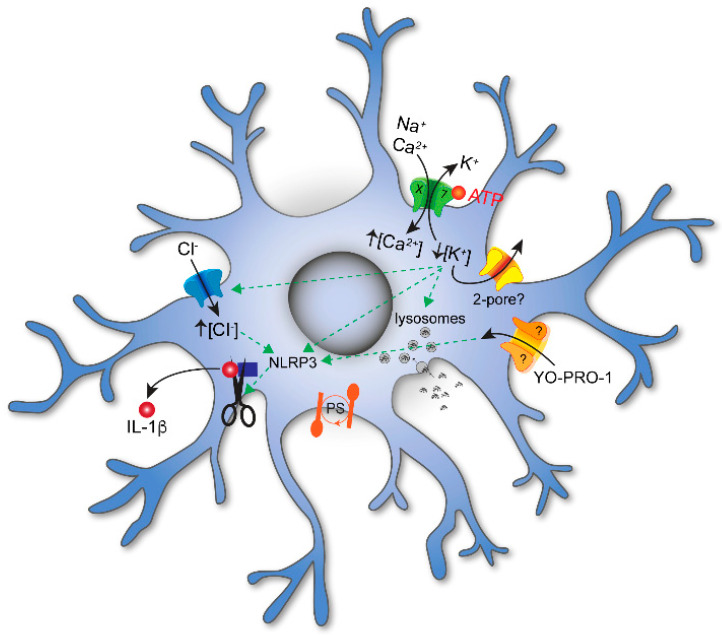
P2X signaling in microglia. Extracellular ATP evokes influx of Na^+^ and Ca^2+^ and efflux of K^+^ through P2X7Rs embedded in the plasmalemmal membrane. ATP-gated efflux of K^+^ is also thought to involve additional channels; at present, the identity of these channels is unknown but may involve 2–pore K^+^ channels. The decrease in [K^+^]_i_ results in influx of Cl^−^ and the maturation and release of the proinflammatory cytokine IL–1β. Activation of P2X7Rs also increases membrane permeabilization of large organic cations like YO-PRO1, decreases phagocytosis, and initiates phosphatidylserine (PS) exposure on the cell surface. While YO-PRO1 permeates the P2X7R pore, other pathways (marked with a “?”) are also thought to play a role.

In contrast, and as expected from work on mice [199], short applications of higher concentrations of eATP (≥1 mM) or the higher affinity ATP analog, 2′,3′-O-(benzoyl-4benzoyl)-ATP (BzATP), evoke inward currents in human microglia with properties expected of a P2X7R-mediated response (Figure 1); the current is cation non-selective, carried in part by Ca^2+^, facilitated during prolonged exposure to agonists, and blocked by P2X7R antagonists [59]. The resting membrane potential of human microglia is unknown. In rodents, it varies with age [200] but averages around −40 mV [19,201]. At this potential, eATP activation of P2X7Rs causes Na^+^ and Ca^2+^ to rush into the cell as K^+^ exits. The net result is membrane depolarization. In rodents, the inward Ca^2+^ current increases the concentration of free intracellular Ca^2+^ ([Ca^2+^]_i_), which triggers cell cycle progression [202], the release of TNF-α [203] and plasminogen [204], activation of the transcription factor NFAT [205], disruption of the cytoskeleton [206], and the production of H_2_O_2_ [207]. At the same time, the outward K^+^ current decreases the concentration of intracellular K^+^ ([K^+^]_i_), leading to activation of the NLRP3 inflammasome and maturation and release of the proinflammatory cytokines, IL-1β and IL-18 [121]. While it is well accepted that the inflammasome activates when [K^+^]_i_ drops below 90 mM [208], more recent evidence suggests that the P2X7R is not the primary K^+^ efflux pathway [209]. Rather, the two-pore K^+^ channels, THIK-1 and TWIK-2, are responsible in microglia [19,210,211] and macrophages [212], respectively. The data supporting a role for THIK-1 and TWIK-2 are convincing, and the conclusions are firm. However, it is unclear why K^+^ efflux through the P2X7R is not sufficient by itself. In the microglial study of Madry et al., the simple fact that 2 mM ATP did not evoke a P2X7R-mediated current is enough to eliminate this receptor from consideration [19]. The lack of response is surprising because others report robust eATP-gated currents with properties unique to P2X7Rs in murine and human microglia [59,199,213,214], perhaps suggesting that P2X7R expression resembles P2X4R expression in its sensitivity to the choice of animal, the activation state of the microglia, and/or the experimental protocols used to study the cell. Regardless, when present, activation of P2X7Rs results in a large efflux of ^86^Rb^+^, a proxy for K^+^, in J774 macrophages and presumably in microglia [215]. Further, eATP promotes recruitment and colocalization of microglial P2X7Rs and NLRP3 to discrete sites of the subplasmalemmal cytoplasm, suggesting that the inflammasome is positioned close enough to directly sense the P2X7R-mediated local drop in [K^+^]_i_ [216].

### 3.1. Membrane Permeabilization and Cell Lysis

Applications of eATP that last longer than a few seconds result in membrane permeabilization, a hallmark property of P2X7R activation [217,218,219]. Permeabilization is the process by which eATP triggers membrane transport of hydrophilic solutes with molecular masses of <900 Da in a direction determined by their electrochemical potential. The process is reversible [32] and does not necessarily lead to cell death. The ability of eATP to permeabilize membranes was first discovered in mouse 3T3 fibroblasts [220], rat mast cells [221], and mouse J774 macrophages [215,222,223], and later described in mouse microglia [224]. Originally thought to be a unique property of P2X7Rs, it was subsequently shown to accompany the activation of purinergic P2X2Rs, P2X3Rs and P2X4Rs [225,226,227] as well as proton-gated TRPV1 receptors [228]. While the physiological consequence of membrane permeabilization is the loss of cytoplasmic components such as ATP, cGMP, glutamate, and spermidine, the underlying process is typically measured as uptake of polyatomic fluorescent biomarkers such as the nucleic acid stains ethidium bromide and YO-PRO-1 [218,219]. For example, in HEK293 cells expressing recombinant P2X7Rs, BzATP triggers an increase in fluorescence that develops slowly over the course of seconds to minutes as dye enters the cell and intercalates DNA [217]. The primary route of entry is unclear [218]. That some of the dye transits the P2X7R pore is convincingly proven using reconstituted receptors and liposomes that lack other proteins [229]. At the same time, eATP and P2X7Rs may activate secondary transport pathways. For example, pannexin-1 is a plasma membrane cation-selective channel related to gap junction proteins that shows a close membrane association with P2X7Rs. Pharmacologically inhibiting pannexin-1 blocks the initial phase of eATP-gated ethidium uptake in human lung alveolar macrophages, demonstrating that it is partially responsible for membrane permeabilization to cationic macromolecules in these cells [230]. The same drugs also block P2X7R-dependent release of IL-1β, suggesting that pannexin-1 is a critical component of eATP-mediated NLRP3 inflammasome activation [230,231]. In contrast, blocking pannexin-1 has no effect on eATP-mediated membrane permeabilization of monocyte-derived human macrophages [232] or cultured human microglia [59], suggesting that local paracrine signaling active in tissue microenvironments may recruit distinct permeabilization pathways in a tissue-specific manner. The hypothesis that eATP gates multiple transport portals is further supported by the finding that, in some cases, P2X7Rs increase membrane permeability to both cations (YO-PRO-1 and ethidium) and anions (glutamate and Lucifer yellow) in the same cell [223,233,234,235]. It is unlikely that large anions travel through P2X7Rs because these channels show a strong preference for cations [217]. In addition, the uptake of cations is temperature-dependent, whereas the uptake of anions is not [236], again supporting the presence of multiple permeation pathways in some cells. With specific regard to human microglia, eATP does not trigger uptake of large anions in cultured cells [59], although this may reflect the downregulation of genes encoding the necessary pathway; additional experiments on cells that more closely resemble the natural phenotype of in situ microglia are needed.

The physiological and pathophysiological outcomes of membrane permeabilization are uncertain [218] and have not been extensively investigated in microglia. As mentioned above, the process is reversible and does not necessarily lead to cell death. For example, a 30 min application of ATP or BzATP causes significant uptake of cationic YO-PRO-1 in human microglia without inducing the release of lactate dehydrogenase, demonstrating that permeabilization is not lytic under these conditions [59]. In contrast, longer incubations result in necrosis or apoptosis in murine microglia, a result that is prevented by pre-incubation of a P2X7R antagonist [43,237]. Cell lysis is blocked by P2X7R antagonists and absent in cells isolated from P2X7R−/− animals, suggesting that the activation of P2X7R is a critical component of the lytic pathway [108]. Interestingly, eATP-activation of P2X2Rs permeabilizes membranes but does not kill cells, suggesting that permeabilization and lysis proceed through different intracellular signaling pathways [226]. If cell death is not the ultimate consequence, then what purpose does permeabilization serve? The recent work of the Grutter laboratory suggests one possibility [227]. Spermidine is a naturally occurring intracellular polyamine that acts at extracellular sites to allosterically modulate ion channel gating [238]. To be effective, it must be secreted. Spermidine permeates multiple subtypes of P2XRs, including P2X7Rs, suggesting that these receptors represent an eATP-dependent egress pathway for polyamines, an effect that may help to explain the ability of P2X7Rs to modulate the activity of neighboring ion channels [219].

### 3.2. Membrane Blebbing and Microvesicular Shedding

Non-apoptotic membrane blebbing occurs when the actin cytoskeleton separates from the plasma membrane; detachment allows the hydrostatic pressure within the cell to push the membrane through the actin cortex, forming an outwardly facing membrane extrusion [239,240]. eATP, working through P2X7Rs, is a potent initiator of non-apoptotic membrane blebbing in many cell types, including human macrophages [232] and murine microglia [13,241,242]. Here, blebbing occurs within minutes of P2X7R activation [241] and reverses when the agonist is removed [232]. As is the case of membrane permeabilization (see above), the physiological sequela of membrane blebbing is uncertain. In tumor cells, blebs facilitate cytokinesis [243], and in streptolysin-permeabilized human embryonic kidney cells, they trap damaged membrane segments and limit further cellular damage [244]. In THP-1 monocytes [241] and primary mouse microglia [13], bleb formation provides a vehicle for IL-1β release. Here, eATP activation of P2X7Rs results in rapid movement of phosphatidylserine and acid sphingomyelinase to the outer leaflet of the plasma membrane, resulting in the formation of small (40–80 nm) membrane-derived microvesicles that contain IL-1β. Surprisingly, eATP does not cause blebbing in cultured human microglia [59].

### 3.3. Cytokines and Reactive Oxygen Species (ROS)

The ability of eATP acting on purinergic P2 receptors to function as a DAMP has long established a role for eATP in regulating neuroinflammatory immune responses [167,245]. The immune response is characterized by a proinflammatory state, which is driven by immune cell activation and the subsequent release of proinflammatory cytokines and reactive oxygen species (ROS) [207,246,247]. In the presence of CNS injury, infection, or neurodegeneration, copious amounts of ATP are released into the extracellular environment from stressed and dying cells [248]. At high enough concentrations, this eATP can promote macrophage and microglial activation, which drives a cascade of P2X7R-mediated events that concludes with the time-dependent release of proinflammatory cytokines, including IL-6, IL-18, TNF-α, and IL-1β [59,247,249,250,251]. Specifically, P2X7R-mediated IL-1β release occurs through a multi-step process that requires priming first by cellular stress or pathogens (i.e., LPS) to stimulate the production of immature pro-IL-1β [121,122,252]. Upon accumulation in the cytosol, pro-IL-1β requires a secondary hit to promote its maturation and secretion driven by caspase-1 activity. Importantly, cells within a quiescent state store caspase-1 in an inactive form (pro-caspase-1), which requires cellular stimulation via DAMPs to undergo maturation to the active form [121]. In the case of eATP functioning as a DAMP, P2X7R activation drives significant K^+^ efflux from the intracellular environment, which subsequently promotes NLRP3 inflammasome complex formation [245,248,252]. The NLRP3 inflammasome is composed of a primary scaffold protein (NLRP3) that recruits the accessory protein ASC, which mediates pro-caspase-1 recruitment and activation [121,245]. Upon activation, caspase-1 drives the cleavage of pro-IL-1β into its active form, which can subsequently be secreted from the cell upon microvesicle shedding from the plasma membrane [13,241]. Importantly, P2X7R activation in microglia also promotes reactive oxygen species (ROS) formation [207,253]. Upon stimulation, P2X7R drives ROS production via p38 MAPK-dependent NADPH oxidase activation [207]. Notably, both the P2X7R-mediated release of proinflammatory cytokines and ROS are highly characterized in the pathophysiology of neurodegenerative diseases [248]. In Alzheimer’s Disease, the P2X7R is significantly upregulated adjacent to the characteristic Aβ plaques where surrounding activated microglial populations are colocalized [207]. Importantly, Aβ stimulates microglial activation, thereby driving the release of proinflammatory cytokines and ROS, which induce pro-apoptotic gene activity, thus mediating the death of neuronal cell populations and exacerbating neuroinflammation [248,254].

### 3.4. Tumor Microenvironment

Tumorigenesis frequently occurs at chronically inflamed tissue sites [255,256,257], where the rate at which the tumor proliferates is largely dependent on a delicate balance of immunosuppressive and immunostimulating cell types coexisting within the tumor microenvironment (TME) [63,191,258,259,260,261]. Underlying components of the TME include stromal cells, fibroblasts, endothelial cells, and infiltrating innate (TAMs, myeloid-derived suppressor cells, dendritic cells) and adaptive (T cells) immune cells [255,262]. Communication between cells occurs through the release of growth factors (VEGF), cytokines (IL-6), chemokines, components of the extracellular matrix, and purines to dictate tumor growth [255,257,263]. Tumor cells express P2X7Rs that drive proliferation by enhancing cellular metabolism and angiogenesis when the concentration of eATP is relatively low [260,264,265]. In contrast, when eATP rises to concentrations ≥100 µM as the result of hypoxic tissue necrosis, eATP promotes tumor cell cytotoxicity through sustained membrane permeabilization [63,260,266]. The ability of ATP to kill cancer cells justifies the development of selective P2X7R agonists as a therapeutic cancer target [266,267].

The immune cells that infiltrate tumors also express high densities of P2X7Rs, which in part determine whether these cells work to promote or eliminate tumor cells [256,268]. P2X7R-driven NLRP3 inflammasome complex activation and subsequent IL-1β release largely account for the immunostimulating qualities of P2X7R. Namely, IL-1β secretion from dendritic cells primes antigen-specific CD8+ T-cells, which release IFN-γ to exert their antitumor effects [256,269]. This tumor-eradicating role is exemplified in cancer models utilizing P2X7R-deficient mice (p2rx7−/−). In the absence of P2X7R, inoculated tumors both proliferated and metastasized at a faster rate compared to those brought up in wild-type mice (p2rx7+/+) [270]. Conversely, P2X7R activation on myeloid-derived suppressor cells fosters tumor-promotion upon the production and release of immunosuppressive factors, including reactive oxygen species, arginase-1, and TGF-β1 [256,263]. Additionally, P2X7R upregulation in glioma-associated microglia drives immunosuppression upon facilitating NLRP3 inflammasome activation and IL-1β release [48,268]. Proinflammatory IL-1β stimulates glioma cells to produce TGF-β, which mediates subsequent upregulation of VEGF, leading to tumor proliferation via increased angiogenesis [271,272,273]. Importantly, eATP also exhibits immunosuppressive effects upon its breakdown to adenosine via ectonucleotidases CD39 and CD73. Particularly, Tregs’ characteristic immunosuppressive activity is based on its high expression level of both ectonucleotidases. Free adenosine can then target P1 A2ARs to inhibit tumor-infiltrating cytotoxic CD8+ T cells [266,272].

### 3.5. Cell Death and Disease

Sustained stimulation with ATP is a potent catalytic stimulus for several cell types, including microglial cells, and the available literature clearly point towards the involvement of P2X7R in ATP-induced cell death, as reviewed by Peter Illes [274]. P2X7R has been described as a death receptor [43,275]. Short periods of P2X7R activation are cytotoxic, and once activated, the P2X7R sets in motion an irreversible death process [276,277]. Cells primed with inflammatory mediators (e.g., lipopolysaccharide) are particularly susceptible to the toxic actions of ATP [278], and this priming effect may alter the distribution or activation of P2X7 receptors in cell membranes [279].

Studies in culture strongly suggest a role for P2X7R-mediated cell death in a number of neurodegenerative diseases. Specific examples include rat microglial cell lines N9 and N13 [237], murine microglial cell line EOC13 [280], mouse primary microglia [207,277], and enteric glia [281].

### 3.6. Oxygen Glucose Deprivation

Oxygen glucose deprivation (OGD) is often used to study ischemic cell death. It negatively impacts microglia motility and induces microglia cell death. Upregulation of the P2X4R and P2X7R is reported to occur in N9 microglial cells deprived of oxygen [282]. Further, the same study suggests that metabolic stress like OGD induces massive release of extracellular ATP, which in turn activates cortical P2X and P2Y receptors. Several P2 receptors (P2X1R, P2X2R, P2X3R, P2X5R, and P2Y11R) alter the homeostatic balance of Ca^2+^ and Na^+^ fluxes, triggering both necrotic and apoptotic pathways [283]. In a similar fashion, P2X4 and P2X7 receptors induce the microglial release of proinflammatory cytokines [246] and subsequent neuronal death. Blocking the receptors with the P2 antagonists PPADS and TNP-ATP reduced microglia activation and rescued cortical cells from OGD-induced cell death [282]. OGD-induced microglial cell death has also been studied in BV2 cells, where the pharmacological inhibition of P2X7R using Brilliant Blue G (BBG) significantly reduced OGD-induced BV2 cell death. Similar results were observed in neonatal hippocampal slices. Here, OGD increases extracellular ATP, and treatments that decreased the concentration of extracellular ATP or reduced the availability of P2X7R receptors inhibited OGD-induced microglia cell death [284]. The depletion of extracellular Ca^2+^ also significantly inhibits cell death, indicating that OGD induces Ca^2+^-dependent microglia cell death [284]. Further in vivo studies performed on a middle cerebral artery occlusion rat model showed that inhibition of P2X7R expression by promoting degradation of ATP protects against the brain injury produced by OGD [285]. However, P2X7Rs are not the sole contributors to the purine- and calcium-dependent ischemic cell death and other mechanisms remain to be discovered.

## 4. Disease States

P2X7Rs are implicated in the genesis and pathology of a number of chronic diseases.

### 4.1. Alzheimer’s Disease

Alzheimer’s disease (AD) is a leading cause of dementia worldwide. No treatments are currently available to stop AD or significantly slow its progression. AD is characterized by cortical atrophy, neuroinflammation, the presence of extracellular senile plaques composed of amyloid beta peptides, intracellular neurofibrillary tangles, neuroinflammation, and the loss of neurons [286,287]. Microglia are implicated in AD, and phenotypic changes in microglia morphology, a hallmark of activation, are seen at the very start of disease progression, and microglia are widely acknowledged as a crucial target of Aβ-dependent toxicity. Further, the microglia of AD patients show an acute proinflammatory response and chronic reduction in phagocytic ability [288,289].

**Figure 2:**
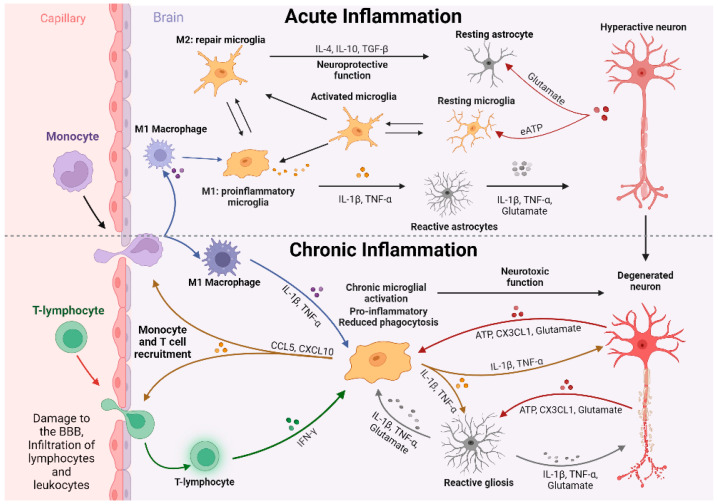
Model for microglial-driven neuroinflammation. Neuroinflammation can be subdivided into acute and chronic phases. Acute neuroinflammation is characterized by activation of resident immune cell populations, release of proinflammatory mediators including cytokines/chemokines, infiltration of peripheral immune cells to assist in the immune response, and phagocytic activity to reduce cellular debris. When inflammation persists past the initial injury event and homeostasis is not maintained, chronic neuroinflammation commences. This phase is characterized by sustained microglial activation and gliosis measured by heightened release of proinflammatory cytokines/chemokines, reduced phagocytosis, and neurodegeneration. Created with BioRender.com accessed on 8 January 2024.

Several studies provide evidence that extracellular ATP plays an important role in ATP-induced neurotoxicity (Figure 2), primarily via the activation of P2X7R [290]. The upregulation of P2X7R has been observed in the microglia surrounding the senile plaques in both human and animal models of AD, such as the Tg2576 transgenic mice, APPswe/PS1De9, J20 mice, and rats following intrahippocampal Aβ injections [291].

The increased activation of P2X7R in microglia and ROS production concentrate in the area of senile plaques appears to increase with age and are parallel with Aβ increase and correlate with synaptotoxicity in AD [292]. In mouse microglia, Aβ increases intracellular Ca^2+^, promotes ATP release, permeabilizes the plasma membrane, and causes an P2X7R-dependent increase in IL-1β [293]. Furthermore, P2X7Rs also regulate Aβ dependent NLRP3 gene transcription, NLRP3 inflammasome activation, and release of IL-1β in microglia [251,294].

In both the in vivo and in vitro models, pharmacological blockade or genetic silencing of the P2X7R prevents activation of microglia and neuroinflammation induced by Aβ [295,296,297,298]. More importantly, in an AD mouse model induced by intrahippocampal Aβ injections, P2X7R also preserves memory and cognitive function by increasing the spine density of hippocampal neurons [299]. Similar effects were also demonstrated in APP/PS1 mice where a lack of P2X7R function led to an increase in synaptic plasticity and improved cognitive abilities [300]. In a familiar Alzheimer’s disease (FAD) model using transgenic P2X7REGFP/J20 mice and human AD patients, it has been shown that P2X7R is upregulated on microglia that surround the senile plaques [301]. Neuroinflammation caused by Aβ increased P2X7R expression on microglia in the advanced and late stages of AD, whereas its expression was found to be decreased in neurons. P2X7R activation enhances the migration of microglia towards senile plaque and reduces its phagocytic capability [301]. The silencing of P2X7R accelerates Aβ phagocytosis by microglia, reduces Aβ load, improves cognitive decline, rescues synaptic dysfunction, and decreases CD8+ T cell recruitment by reducing chemokine production [300].

The accumulation of Aβ also leads to the impairment of mitochondrial oxidative phosphorylation by altering complex IV, F0F1 ATP synthase and mitochondrial permeability transition pore (mPTP) [302,303,304]. P2X7R-deficient mice are resistant to Aβ dependent mitochondrial hyperpolarization and cyt c release [294]. The Aβ-dependent toxicity of microglia relies on the ability of P2X7R to promote cellular uptake of Aβ through innate phagocytosis and its localization to mitochondria. Thus, in isolated and permeabilized mitochondria, the lack of P2X7R was not able to prevent the inhibition of F0/F1 ATP synthase by Aβ. Further, this study showed that the Ca^2+^ channel blocker, nimodipine, strongly reduced all the Aβ induced effects, such as NF-κB activation, NLRP3 inflammasome stimulation, cyt c release IL1β release, mitochondrial dysfunction and cell death. Nimodipine did not exert its effect by directly antagonizing the P2X7R, but it prevented the delivery of Aβ to the mitochondria; thus, it might be possible that nimodipine targets a pathway downstream from the activation of P2X7R [294].

The 489C > T polymorphism in the P2X7R gene decreases the probability of having AD as this polymorphism alone or in concomitance with 1513A > C polymorphism is less frequent in AD patients compared to age-matched non-demented elderly [305]. A recent study performed on 5X FAD mice suggested that taurodeoxycholate (TDCA), a GPCR19 ligand, can reduce neuroinflammation in AD patients. In their study, TDCA was found to inhibit the priming phase of NLRP3 inflammasome activation, the suppression of P2X7R expression and P2X7R-mediated calcium mobilization, all of which are necessary for the production of IL-1β/IL-18 from microglia [306]. Transvagal nerve stimulation can be another promising strategy to improve spatial memory and learning in APP/PS1 mice by inhibiting hippocampal P2X7R/NLRP3/Caspase-1 signaling; however, more studies are required to establish its role in improving cognition [307]. Altogether, these studies suggest that modifying the microglia function or targeting the P2X7R may be a promising strategy in the therapeutic management of AD.

### 4.2. Parkinson’s Disease

Parkinson’s disease (PD) is a progressive nervous system disorder characterized by the loss of dopaminergic (DA) neurons in the substantia nigra and the presence of intra-neuronal cytoplasmic inclusions containing α-synuclein and neurites. Clinically, PD symptoms include bradykinesia, postural instability, resting tremors and muscle rigidity. In animal models of PD, such as 6-OHDA, MPTP and rotenone, and PD patient’s microglial activation and chronic neuroinflammation, have been shown to contribute to the degeneration of DA neurons in the striatum and substantia nigra [308,309]. The increased expression of P2X7R has been associated with PD and contributes to the pathogenesis of PD by affecting gliosis, synaptotoxicity and neurotoxicity [310,311,312]. The accumulation and oligomerization of misfolded α-synuclein named Lewy bodies has been recognized as a central player in the pathology of PD. In BV-2 cells and primary culture microglia, α-synuclein binds and activates P2X7R in microglia, activating the PI3K/AKT pathway and inducing ROS production [313]. This leads to deregulation in dopaminergic and glutamatergic synaptic transmission, causing neurotoxicity. α-synuclein also stimulates P2X7R transcription, which might be one of the reasons for the observed upregulation of P2X7R in PD patients. In mice, increased expression of P2X7R was observed with the acute 6-OHDA toxin model in a time-dependent manner, which coincides with an increase in translocator protein (TSPO), leading to increased binding of P2X7R with TSPO and motor behavior changes. Chronic α-synuclein model leads to an increase in TSPO without altering P2X7R expression, suggesting that increased P2X7R binding with TSPO is associated with neuroinflammation in acute but not chronic rodent models of Parkinson’s disease [314]. This study suggests that an alternative mechanism might play a role in inducing neuroinflammation relating to α-synucleinopathy in rodents. The microglia recognize, uptake and phagocyte α-synuclein. In rat LPS models and 6-OHDA models of PD, P2X7R antagonist BBG has been shown to reduce damage to dopaminergic neurons, attenuate LPS-induced upregulation of the expression of P2X7R, microglial activation, mitochondrial dysfunction and behavioral deficits [315,316]. Uncontrolled microglial activation in PD secretes Cathepsin L (CTSL) from microsomes, inducing neuronal damage and death. This study identified the P2X7R/PI3K/AKT signaling pathway as the underlying molecular mechanism [317]. The P2Y6 receptor has also been shown to have a potential role in the pathogenesis of PD. Apart from P2X7R, an increased level of P2Y6R has also been shown in the PBMCs of sporadic PD patients, suggesting that it is a potential clinical biomarker [318]. Both the in vivo and in vitro studies performed on the 6-OHDA model of PD have shown the neuroprotective effect of P2X7R and P2Y6 antagonists [319]. Thus, P2X7R and P2Y6R antagonists may have therapeutic potential in terms of PD.

### 4.3. Epilepsy

Epilepsy is a prevalent neurological disease that afflicts more than 70 million people worldwide [320], often presenting with cognitive, behavioral, and psychological abnormalities [321]. Current anti-epileptic drugs (AEDs) focus on a neurocentric approach to pharmacological intervention, thereby targeting the classical seizure model fixated on hypersynchronization of neuronal output and an imbalance in excitation/inhibition coupling [322,323]. However, thirty percent of epilepsies are completely refractive to AEDs [324], highlighting the importance of developing therapeutics with novel mechanisms of action. Importantly, recent studies suggest a strong contribution of neuroinflammation and neuroglia in the pathophysiology of epilepsy [325]. Specifically, translational work in mice and rats suggest that P2XRs could be novel targets for AED development due to epilepsy promoting the upregulation of P2Y6, P2Y12, P2X4, and P2X7 receptors on activated microglia [326,327] and promote neuroinflammation. Specifically, Wang et al. recently showed that usage of the anti-inflammatory drug Astaxanthin can attenuate the P2X7R-mediated neuroinflammation that is associated with status epilepticus (SE) [328]. As outlined above, limited release and rapid degradation limit the amount of ATP in the extracellular milieu of healthy brains. However, the concentration increases in response to the excessive neuronal firing that characteristically occurs throughout an epileptic seizure [326,327,329]. The result is a chronic neuroinflammatory state that disrupts the integrity of the blood–brain barrier, permitting infiltration of peripheral immune cells such as brain-infiltrating leukocytes into the brain, driving further inflammation [330,331]. A study performed using a lithium-pilocarpine-induced epileptic rat model demonstrates that increased expression of P2X7R in activated microglia promotes depression and anxiety-like behaviors in epileptic rats. P2X7R antagonist BBG reversed these effects as effectively as the classic anti-depressive and anti-anxiety drug fluoxetine [332]. Another study based on a similar animal model for status epilepticus (SE) revealed that protein disulfide isomerase (PDI)-mediated redox/S-nitrosylation may be responsible for facilitating the trafficking of P2X7R to the cell surface. P2X7R further promotes microglial activation and astroglial apoptosis following SE as L-NAME and PDI siRNA-attenuated SE-induced microglial activation and astroglial apoptosis [333]. In palmitoyl protein thioesterase 1 (PPT1) knock-in mice, astrocyte activation precedes microglial activation and neuronal death. The decreased expression of synaptic protein GluN2B and GABAARα1 in the hippocampus of PPT1-KI mice was observed, along with the activation of P2X7R in the microglia, causing the release of proinflammatory cytokines IL-1β and TNF-α. The cytokines further act on neurons and astrocytes, releasing ATP and glutamate, leading to increased neuronal excitability and seizures [334]. Such a scenario suggests that targeting P2X7R-mediated microglial activation and the subsequent release of proinflammatory cytokines may limit epilepsy-associated neuroinflammation, providing a novel mechanism of action to treat drug-resistant epilepsy. Treatment with P2X7 receptor antagonists has also proven to be beneficial in hypoxia-induced neonatal seizures and the subsequent development of epilepsy [335].

## 5. Conclusions

While microglial P2X7Rs undoubtedly play key roles in the formation, maintenance, and pathology of the CNS, our understanding of how this neuromodulation occurs is incompletely understood. This is particularly true of the actions of eATP on human microglia, for which an accessible and reliable in vitro experimental model is lacking. Future studies of human microglia maintained in a milieu that accurately replicates the CNS microenvironments are badly needed. Until then, we are left to rely on increasingly sophisticated animal models that regularly highlight the importance of microglia, eATP, and P2X7Rs in initiating and modulating CNS function.
